# Germ Granules in Animal Oogenesis

**DOI:** 10.3390/jdb10040043

**Published:** 2022-10-09

**Authors:** Mikhail A. Dobrynin, Ekaterina O. Bashendjieva, Natella I. Enukashvily

**Affiliations:** The Laboratory of Non-Coding DNA, Institute of Cytology of the Russian Academy of Sciences, 194064 St. Petersburg, Russia

**Keywords:** oogenesis, embryogenesis, germ granules, nuage, membraneless biomolecular condensates

## Abstract

In eukaryotic cells, many macromolecules are organized as membraneless biomolecular condensates (or biocondensates). Liquid–liquid and liquid–solid phase transitions are the drivers of the condensation process. The absence of membrane borders makes biocondensates very flexible in their composition and functions, which vary in different cells and tissues. Some biocondensates are specific for germ line cells and are, thus, termed germ granules. This review summarizes the recent data on the composition of germ granules and their functions in gametes. According to these data, germ granules are involved in the determination of germline cells in some animals, such as *Amphibia*. In other animals, such as *Mammalia*, germ granules are involved in the processes of transposons inactivation and sequestration of mRNA and proteins to temporarily decrease their activity. The new data on germ granules composition and functions sheds light on germ cell differentiation and maturation properties.

## 1. Introduction

Several forms of macromolecules compartmentalization are established in eukaryotic cells. Some compartments (e.g., nucleus, mitochondria and endoplasmic reticulum) are surrounded by lipid membranes. Another option is the organization of macromolecules as bimolecular condensates (biocondensates). These are membraneless compartments, with their assembly being induced by a high local concentration of certain proteins and nucleic acids. Phase transition processes in these areas play a key role in the biocondensates assembly [1,2,3].

In germ line cells, specific biomolecular ribonucleoprotein condensates, termed “germ granules” (GG) or “germinal cell granules”, have been identified. The first description of membraneless intracellular granules was provided by E. Metschnikoff [4]. Diffusely distributed GG components can aggregate into liquid drops during liquid–liquid phase transitions or “solid” bodies during liquid–solid state transitions, due to reversible polyvalent interactions. 

Biomolecular condensation occurs via the liquid–liquid phase separation of intrinsically disordered proteins/regions (IDPs/IDRs), along with other biomolecules (DNA, RNA etc). Depending on the physicochemical conditions, the molecules can condensate into liquid droplets, gels, or solid-like granules, where “solid” is a viscoelastic macromolecular precipitate consisting of precipitated protein [1,5]. Despite the large quantity of data, no consensus has been reached regarding the nomenclature, composition, and functions of GG. The absence of a consensus complicates the description and interpretation of results, as well as the communication between different research groups. The purpose of this review is to summarize the latest data on GG and their proposed functions. 

## 2. Inductive and Inherited Determination of Germ Line Cells

Cells that form the germline differ from somatic cells in a number of specific functional features. The somatic differentiation is blocked in germline cells, but the capacity to form a totipotent zygote after fertilization is preserved. Their main function is to form a pool of primordial germ cells (PGC) in the developing gonad. There are two main mechanisms of germline determination: inherited and inductive. Inherited (or ‘preformative’) determination relies on the inheritance of the maternal factors accumulating in oocytes. These factors are the basis for the formation of a structure referred to as ‘germplasm’ (GP) (Table 1).

The GP was originally identified within the oocyte as an electron-dense material [25]. It contains important maternal RNAs and the proteins needed for oogenesis and embryogenesis, as well as for the preformative determination of germ cell lines in many species. Most germ plasm RNAs are related to cell fate determination, such as GC. Many GP proteins are members of the piRNA pathway, which protects the integrity of the germline genome by attacking transposons RNA [25]. The GP is a substrate for forming GG, which are referred to differently in various species. GG are termed ‘nuage’ (French for “cloud”), when located in the perinuclear area [26]. GG are large, non-membrane-bound, ribonucleoprotein (RNP) organelles found in the germ line cytoplasm of most, if not all, animals [27]. Nowadays, the term ‘nuage‘ is currently used with lesser frequency because of its ambiguity in the description of perinuclear GG that are very heterogeneous in their composition and functions. Cells that inherit GG eventually become PGCs. This mechanism is typical for insects, such as *Drosophila* [9], as well as some vertebrates, such as *Danio* and *Xenopus* [14,28]. 

Some of the germplasm proteins that have been identified are specific for the animal or vegetal pole of the oocyte and zygote of the ‘pre-formative’ species. Animal pole germplasm factors include Vasa protein (DEAD-box RNA helicase 4 or DDX4 in mammals). *Vasa* was originally identified in Drosophila as a gene required for abdominal segment formation and germ cell detection [29]. It has since been identified in the germ cells of a wide range of animals, from sponges and flatworms to birds and mammals. Vasa is the most widely used germ cell lineage marker [30]. In animals with inherited germ cell specification (including insects, nematodes, anurans, and birds, but not mammals and caudal amphibians), germ cell fate is determined by germplasm inheritance, rather than cell signalling. In these species, DDX4 is localized in the germplasm and can be used to track a germ cell line from its first appearance (often during the first few cleavage divisions) to adulthood [31].

DDX4, similar to many proteins of the DEAD-box family, has ATP-dependent RNA-helicase activity and RNA-dependent ATPase activity [30]. The DDX4 helicase uses the energy of ATP to separate the base pairs of the RNA double strand, for example, to make the RNA available to a nuclease. The presence of DDX4 as an obligatory component of germ cells is associated with the physicochemical characteristics of this protein. It has been shown in vitro and in vivo that DDX4, similar to other members of the DEAD-box family, can undergo liquid–liquid phase separation (LLPS). LLPS occurs due to temporary multivalent interactions (electrostatic, hydrophobic, π-cationic, cation-cationic, and π–π-interactions between neighboring molecules), thus forming membraneless RNP biocondensates, whose contents are isolated from other areas of the cell [1,2]. Through RNA-dependent ATPase activity, DDX4 regulates RNP phase separation: DDX4, by binding ATP, forms multivalent interactions with RNA to promote phase separation. DDX4-ATPase activity is controlled and can be activated by specific cofactors. ATP hydrolysis triggers RNA release, thereby disrupting multivalence and, as a result, causing the disassembly of RNA-containing membraneless organelles [3].

Proteins of the Nanos family that are expressed in pluripotent cells, stem cells, and/or germ cells are an important GP component in many species, including human [32]. The family members are distinguished by two CCHC zinc fingers that interact with RNAs in a non-sequence-specific fashion. Nanos functions as a translational repressor, but it requires Pum (Pumilio protein) to confer RNA-binding specificity [33]. Nanos homologs in the *Caenorhabditis elegans, Drosophila, Danio, Xenopus*, and *Mus* genera have been shown to perform essential functions in maintaining germ cell fate and survival [34,35,36,37]. The number of expressed nanos genes differs between species, e.g., one gene is expressed in Xenopus sp or Drosophila sp, while in Mus sp, Homo sapiens, three nanos genes are activated [37].

Multi-tudor domain-containing proteins (Tdrds) play important roles in the formation of nuage. They are involved in the piRNA pathway [38]. Drosophila tudor (Tud) has been shown to interact with Piwi proteins Au and Ago3 and plays a role in the localization of Aub to Gp and polar granule formation [39]. In mice, TDRD6 plays a role in establishing the chromatoid body and localization of piRNA pathway components to this body [40]. Additionally, Tdrd6a is required for the coordinated loading of essential GG components into PGCs, through fine-tuning of the aggregating properties and mobility of the Bb organizer Buc in zebrafish.

Proteins of the Dicer family play an important role in the formation of GG/nuage. These proteins are required for the miRNA and siRNA pathways of many animals [7,41,42]. It has also been found that Dicer proteins can interact with Vasa [42].

Vegetal pole germ plasm RNPs contain RNAs encoded by deleted in azoospermia-like (*dazl*) and bruno-like (*brul*) genes. In *Danio rerio*, *dazl* RNA aggregates are deposited in the vegetal cortex in the mature egg and move animally after egg activation. At 45 min post-fertilization, *dazl* RNP aggregates begin to be observed at the site of germplasm accumulation at the first cleavage furrow [43]

The second mechanism of determination engages inductive mechanisms. That means that cell fate is defined not by the deposited maternal components, but rather it is induced by signaling events from the surrounding cells during embryo development. The inductive mechanism is attributed to all mammals, including mice and humans [44,45]. The switch between the two mechanisms occurred many times during evolution. It has been suggested that the switch is not a ‘yes or no’ event (‘inherited or inductive’), but a continuum of different combinations of both pathways [46].

## 3. Diversity of RNP Granules in Germline Cells

GG are a heterogeneous mix of RNA and protein. These organelles are found in the germline cytoplasm of almost all studied animals from *Caenorhabditis elegans* to human (Table 1).

### GG Nomenclature Problems

GG are highly dynamic during germline development. They often change their size, morphology, and association with other organelles. The functions of GG can differ in different species and at different stages of gametogenesis. Therefore, similar structures have been repeatedly described in different animals under different names (Table 1). Nowadays, due to the detection in GG of a wide range of RNAs and proteins [9,14,20,47], some sets of molecular markers have been identified that allow a more reliable classification of these structures (Table 2). However, depending on the stage of germ cell development, GG RNA and proteins can change their localization and functions: they are either translationally repressed before fertilization or diffusely distributed throughout the cytoplasm.

## 4. P-Granules in *Caenorhabditis elegans*

**Figure 1:**
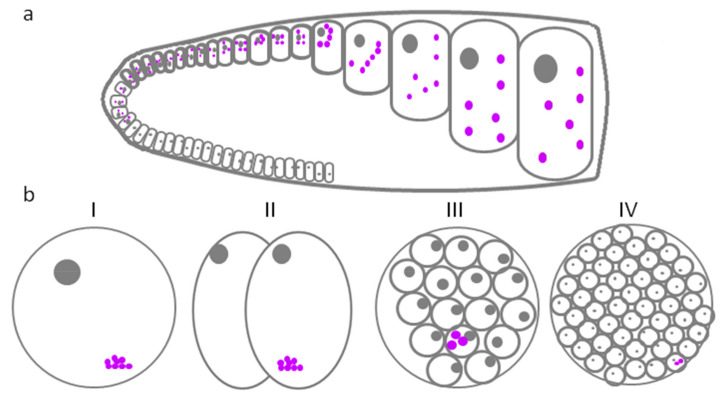
P-granules of *C. elegans*. (**a**) Schematic diagram of a longitudinal section through one arm of an adult hermaphrodite gonad. The drawing represents the P-granules localization at different stages of oogenesis. Spermatogenesis is not shown. (**b**) Localization of P-granules at the early stages of embryogenesis (I-zygote, II-2-cells embryo, III-16-cells embryo, and IV-in an embryo with formed germline cells (Z2 and Z3)). Cells are outlined with grey, and nuclei are shown as closed grey circles. P-granules—closed purple circles.

GG in *Caenorhabditis elegans* were originally named P-granules, obtaining their name from the cell line where they were found (P-lineage cells) [6,108]. P-granules are localized near the nuclear membrane during almost all of the development of the germ cells. However, during oocyte maturation, they are distributed throughout the cytoplasm [6]. Later, after fertilization, maternally contributed GG in the 1-cell zygote (P0) are progressively segregated to the germ line blastomeres, or P cells (P1, P2, P3, and P4), through four asymmetric cell divisions, thus resulting in the delivery of germ granules to the P4 cell, which is the *C. elegans* PGC (Figure 1) [109,110]. In newly fertilized embryos, P-granules are concentrated at the vegetative pole, creating an asymmetry; after the first division, they are inherited by the blastomere that will become the PGC progenitor. In PGC, granules are scattered throughout the cytoplasm, until the embryo reaches the eight-cell stage, when they are relocated toward the nuclear membrane (Figure 1). When PGCs begin to assemble, P-granule protein components, such as ZNFX-1 and WAGO-4 separate to form adjacent exogenous siRNA processing centers, called Z granules and mutator foci (respectively), which persist as separate phases in the adult germline [52]. In the eight-cell embryo, P granules are restricted to P3 cells. P3 will undergo asymmetric division to produce the D and P4 cells, to which P granules are segregated. All P-granules continue to segregate asymmetrically and reside in only one blastomere during embryo cleavage [111]. P-granules are a specific feature of germline cells. They can even be used as markers, when establishing the three principal body axes of *C. elegans* [111].

P-granules reside close to organelles, such as the mitochondria [112,113]. However, mitochondria, microtubules, and centrioles are rarely detected inside the GG. Mitochondrial RNA is not a prominent component of P-granules. It is believed mitochondria can get into GG accidentally, during the formation of a granule or when they fuse [48,112]. Some of the proteins and RNAs residing in P-granules have been identified. Four DEAD box paralogs of Vasa RNA-helicase, termed as germline helicases GLH: GLH-1, GLH-2, GLH-3, and GLH-4 and helicase RDE-12 were described [53,54]. In the same way as in the mammalian mouse homologue Vasa, MVH, the glycine-rich GLH domains of *C. elegans* are built on phenylalanine—glycine (FG) repeats, instead of the arginine, and glycine (RG) repeats are found in most other Vasa orthologs. The RG domain, in contrast, is found in a germ-line specific protein PGL-1 and PGL-3 [53,55]. In addition, the RG domain was found in some proteins, which associate with P granules at least transiently: in DEAD-box helicases LAF-1 and VBH-1, LSM14 family protein CAR-1, and Argonaute proteins CSR-1, ALG-3, ALG-4, and HRDE-1. [56] RG repeats are RNA-binding domains [114], whereas FG repeats bind to nuclear pore components [115]. In *C. elegans*, FG-nucleoporins are necessary for retaining P-granules association with nuclear pore complexes [56]. P granules, similar to nuclear pore complexes, are held together by weak hydrophobic interactions and establish a size-exclusion barrier [55]. PGL-1, GLH-1, GLH-2, GLH-3, and GLH-4 are involved in the regulation of translation and found in P-granules at all stages of the life cycle of *C. elegans* [47,53,116]. Most nuclear pores (75%) in germ line cells are associated with P-granules [48]. The FG domains of GLH proteins probably contribute to the localization of P-granules near the nuclear surface. Nevertheless, GLH-1 and its FG domain are not sufficient to form granules, but require factors such as PGL-1 to nucleate the localized concentration of GLH proteins [55]. P-granules also contain proteins of the MEG family, i.e., MEG-1, MEG-2, MEG-3, and MEG-4, which belong to the third type of IDR, consisting of a long, serine-rich N-terminus [58]. MEG-3 and MEG-4 also contain an HMG-box in their C-terminal tail and share some homology with the ancient GCNA family of disordered proteins that function in the germline [117]. All four MEGs are expressed in the early embryo and required for proper germline development [118]. Intrinsically, disordered MEG-3 is required for mRNAs recruitment into P-granules via condensation. MEG-3 traps mRNAs into non-dynamic condensates in vitro and binds to ~500 mRNAs in vivo in a sequence-independent manner. Some of these mRNAs encode germ cell fate regulators [119]. Nanos translation repressors were found in P-granules [35], along with an RNA-helicase CGH-1 [59], translation regulator IFE-1 (eIF4E) [60], and RNA processing protein DCP-2 [61]. Proteins involved in the regulation and synthesis of miRNA/piRNA, i.e., CSR-1 and PRG-1, and proteins of the Dicer family (Dicer-related helicase, DRH-3, and DCR-1), which are directly involved in the formation of miRNA, were also identified among the P-granules components [50,51]. Mutations in *prg-1* cause a significant reduction in the level of a subset of mRNAs expressed during spermatogenesis, and a sterile phenotype was formed, due to defects in spermatogenesis. PRG-1 promotes expression, processing, or stability of piRNA (21U), which, in turn, or in concert with PRG-1, promotes proper expression of spermatogenesis transcripts [50]. 

P-granules apparently play a role in mRNA processing [110,120]. One of the other functions of P-granules is the inactivation of transposons via the piRNA pathway. At the same time, P-granules protect germline transcripts from inactivation via piRNA-initiated silencing [121].

P-granules were the first membraneless organelles subjected to analysis of their physical and chemical properties. The studies were based on the new data of condensed matter physics and polymer chemistry. It was found that P-granules assembly is driven by phase transition mechanisms [62]. During the process, the arginine-rich sequences of proteins with IDR undergo a liquid–liquid phase separation, due to polyvalent electrostatic interactions with RNA, forming liquid droplets (RNP granules). Droplets can flow, bend around the surfaces of other structures, and divide. Condensates formation depends not only on the presence of IDR proteins, but also on their molecular assembly and internal solubility. For example, the solubility of one of the P-granule components, PGL-3 protein, depends on the MEX-5 and PAR-1 proteins [62]. The local concentration of IDR proteins also plays a role in condensation. This finding means that any mechanism that changes the local concentration of the key components, including changes in the expression, degradation, and localization of IDR proteins and regulatory proteins, will affect the formation and volume of the condensed phase. Among these mechanisms, post-translational protein modifications, such as phosphorylation, have been found in *C. elegans* [58]. 

Thus, the main properties of *C. elegans* P-granules are the dynamic variability of localization, composition, and physical properties, as well as a high content of helicases and factors involved in RNA processing. The main functions of the granules are the determination of the germ line cells by depositing proteins and RNA molecules that are important for development. P-granules are also involved in the storage, processing, and degradation of a number of mRNAs, as well as the regulation of piRNA/miRNA and endo-siRNA.

## 5. Drosophila GG

**Figure 2:**
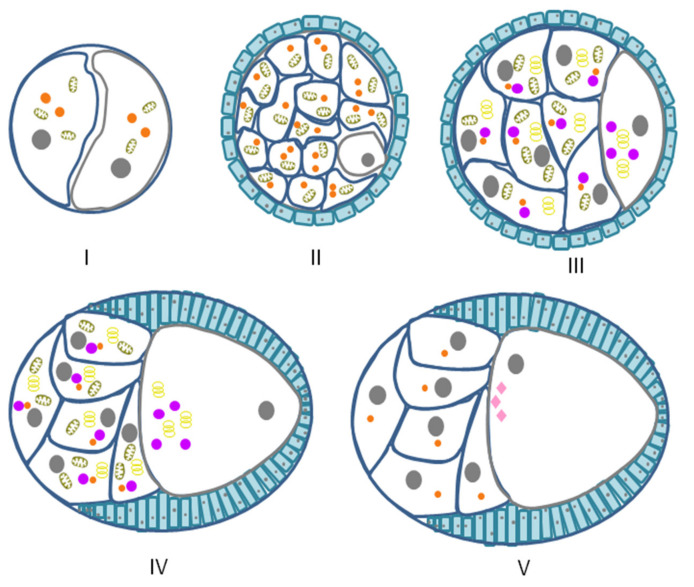
Schematic diagram of various stages of *Drosophila* oogenesis. (**I**). Schematic drawing of the germarium. (**II**). Stage 2, the daughter cell divides incompletely to produce a cyst of 16 cells. One of the 16 cells becomes an oocyte (grey outline), and the other 15 differentiate into nurse cells (blue outline). (**III**). Stages 6–8, the maturing cyst moves down the germarium getting surrounded by somatic follicle cells (blue fill), which intercalate and pinch it off to form a discrete egg chamber. The first polarization of the oocyte is shown. (**IV**). Stage 9. (**V**). Stage 10, stem cells are outlined with grey, daughter cells are outlined with blue, follicular cells are outlined with jade green, and nuclei are shown as closed grey circles. Sponge bodies (closed purple circles). Nuage (closed orange circles). Polar granules (closed pink rhombs). Mitochondria (brown ellipse). Golgi (yellow rings).

Many GG that are built on RNP have been described in *Drosophila* [122]. They are found in two types of cells—in oocytes and in nurse cells that surround them (Figure 2). In *Drosophila*, each of the two ovaries contains ovarioles (chains of egg chambers), the front parts of which are connected to the germarium—the region where germinal stem cells are located. When a female germinal stem cell divides, the daughter cell that lies closer to the anterior part of the germarium retains stem cell identity; the other cell undergo differentiation as a cystoblast. The cystoblast and its progeny undergo four rounds of cell division with incomplete cytokinesis, i.e., the cells remain connected to each other by cytoplasmic bridges. Of the 16 germ cells, only one will become an oocyte; the other 15 cells will become nurse cells [123]. Bridges pass through the structures associated with the cytoskeleton—ringcanals (Figure 2) [124]. The function of nurse cells is to generate the rRNA, proteins, and other components required for early development and deposit them in the growing oocyte [125].

In *Drosophila*, germ cells contain GG that differ in their morphological criteria and subcellular localization. The term ‘nuage‘ has historically been used to describe small GG found near the nuclei of nurse cells. The GG found both in nurse cells and oocytes were referred to as sponge bodies. Large GG at the vegetal pole of late-stage oocytes and embryos have been given the name ‘polar granules’ [8,64,126]. All these RNP differ in their molecular composition (Table 2).

### 5.1. Sponge Bodies

Sponge bodies were first described as an electron-dense, membraneless, mitochondria-associated structure in nurse cells and oocytes [8]. The structure was further described as endoplasmic reticulum-rich structures that lack ribosomes, but contain many components that are also found in P-bodies. The authors also demonstrated that the sponge bodies are not identical to the Golgi complexes. They suggested that the sponge bodies are homologous to the mitochondrial cloud (‘nuage’) in *Xenopus* oocytes, a granulo-fibrillar structure that contains the RNAs involved in the patterning of the embryo. Sponge bodies surround nuage, a possible polar granule precursor. The composition of sponge bodies differs between the nurse cells and oocyte. DEAD-box RNA helicase Me31B and exuperantia, a protein required for localization of the *bicoid* RNA (a product of the homeotic gene Bicoid, a morphogen that determines the development of the acron, head, and thorax of the *Drosophila* embryo), were found in both types of cells [8,63,64]. Bruno protein is concentrated in nurse cells, whereas Orb protein is more typical for oocytes [8,64,65,66]. In nurse cells, sponge bodies appear to surround the nuage particles that have been implicated in the genesis of polar granules in the oocyte [113]. Sponge bodies can move through the ring canals between nurse cells, as well as from nurse cells to oocytes. This confirms the hypothesis that the difference in the composition of the sponge bodies in nurse cells and oocytes is associated with transport functions [65,66]. Based on the study of the composition of sponge bodies, two assumptions were made regarding their functions. According to the first hypothesis, sponge bodies are involved in the post-transcriptional regulation of gene expression during embryogenesis. It is assumed that sponge bodies in *Drosophila* are similar to the P-granules of nematodes [68].

Differences in the morphology of P-granules and sponge bodies are arguments against this point of view. There are hypotheses that these structures can represent different levels of the GG hierarchy [65,66]. Supporters of the second theory believe that sponge bodies act as P-bodies (processing bodies). P-bodies, in this context, should be distinguished from the above-described P-granules of *C. elegans*. P-bodies are aggregates of highly conserved RNPs that are found in both somatic and germ cells. It has been established that such P-bodies are involved in the degradation and storage of RNA, and they may be in the repression of their translation [127]. Proteins of P-bodies were found in sponge bodies: translation initiation protein EIF4e [64] and the Cup protein that binds it [67], mRNA-decapping enzyme 1 Dcp1 and Dcp2 [68]. Further research is necessary to understand the connection between *Drosophila* P-bodies and GG. 

### 5.2. Polar Granules

Polar granules make up the germplasm at the posterior pole of the mature egg (and the early embryo), where they are obtained from nurse cells [128]. It is known that the Oskar and Vasa proteins are essential for the assembly of polar granule components [70,71]. Once *osk* RNA reaches the posterior pole, it is translated into two protein isoforms: long and short Oskar. Long Oskar localizes to the oocyte and embryo posterior cortex and is associated with endosomes, while short Oskar is an integral part of the polar granules [128]. Additional components are proteins Dcp1 and Me31B [64,72]. The presence of Dcp1 and Me31B demonstrates the link between the polar granules and P-bodies [129]. The granules also contain a protein of the Argonaute–Aubergine family [73], which is involved in the regulation of miRNA/piRNA activity. Later, proteins of the PIWI family [41], as well as proteins of the translation regulators of the Tudor [130] and EIF4A [64] families, were identified in these structures, indicating the participation of polar granules in the processes of post-transcriptional regulation of embryonic development genes expression.

Real-time studies using fluorescent GFP-Aubergine protein confirmed that polar granules were assembled in the oocyte de novo. Polar granules incorporate new components that are slower than nuage, suggesting that polar granules may be less dynamic RNP structures, as compared to nuage [65].

### 5.3. Nuage

Nuage is an electron-dense perinuclear structure in *Drosophila*. The term ‘nuage‘ is often used alongside other terms for perinuclear GG in different animals. For example, a suggestion has been made regarding some parallels between nuage in *Drosophila*, as well as nuage in mouse, human, and perinuclear P-granules in *C. elegans* [14,21,48,99,105]. *Drosophila* nuage components include the Vasa protein [70], proteins of the Argonautes subfamily: Aubergine [73], Ago3 [76], the Maelstrom protein, and the Spindle-E protein [77]. While Oskar is, in general, critical for the assembly of germplasm, it is not needed for nuage and absent in the structures [131]. Spindle-E controls the perinuclear localization of two other nuage components: Vasa and Maelstrom [77,132]. Aubergine and Maelstrom are involved in suppressing LINE expression by small interfering RNAs (siRNAs) [75]. Proteins containing the Tudor domain, both Tudor itself [74] and the proteins Krimp, Tejas, and PAPI [75,78,79], have also been identified in these membraneless condensates. The function of the Tudor-domain containing proteins is to attract methylated proteins to GG and recruit mitochondrial and ribosomal RNA into polar granules. Nuage can be observed, since the formation of primary germ line cells, and it is preserved in all germ line cells until an oocyte is established [9]. At the later stages, nuage can be found in all nurse cells. The Vasa protein, one of the nuage marker proteins, was found in second-type perinuclear bodies and polar granules in the oocyte of another insect, the scorpion-fly *Panorpa communis* [133]. The second-type perinuclear bodies of *Panorpa communis* are considered as homologues of the nuage of *Drosophila* nurse cells. Thus, nuage is a conservative structure that exists in germ cells of many species. Beside the Vasa protein, uridine-rich small nuclear RNAs (U-snRNAs) were found in the cytoplasmic perinuclear bodies, which are involved in the key stages of pre-mRNA processing in the nucleus of eukaryotic cells [133]. Later, U-snRNAs, in a complex with core splicing proteins Sm/Lsm, motor neuron survival protein SMN, and *Drosophila* arginine methyltransferases Dart 5 and Dart 7, were found in the cytoplasm of *Drosophila* oocytes and nurse cells. They were described as structures that are now referred to as “U-bodies” [134]. The level of SMN expression in germ line cells is higher than in somatic cells. The deposition of the protein is necessary for its subsequent use during embryogenesis [135].

U-snRNAs are transcribed in the nucleus and exported to the cytoplasm, where they acquire the trimethylguanosine cap and bind to Sm/Lsm proteins. RNP particles accumulate in the U-bodies in the cytoplasm before being imported into the nucleus. U-bodies are spherical and do not colocalize with any known cytoplasmic organelles, except P-bodies, with which they are closely related. Some stages of U-snRNP assembly, perhaps, require the exchange of molecules between these structures [136]. It is possible that the association of U and P-bodies may reflect a feedback mechanism that supports the regulated release of snRNP from U-bodies, depending on the rate of mRNA degradation in P-bodies. Alternatively, the assembly/storage of snRNP in U-bodies can be counterbalanced by the degradation of snRNP in P-bodies. At the same time, mutations of the U-body component—the SMN protein—cause a disruption of the P-bodies; vice versa, mutations of the P-body components—the Cup and Otu proteins—lead to an abnormal distribution and size of the U-bodies [135]. Apparently, the U- and P-bodies are interdependent, and the functions of one of them can be regulated by the mechanisms of the other body.

Thus, several types of GG have been identified in *Drosophila*. Their functions are different in different cells of the germline. The presence of the proteins Me31B, EIF4e, Cup, and Dcp1 indicates the involvement of sponge bodies in the processes of RNA processing. The presence of Bruno and Orb proteins indicates the participation of sponge bodies in the transport of biomolecules between the different types of cells. *Drosophila* nuage is a depot of developmentally important proteins and involved in retroposones inactivation. Polar granules are involved in the translational control of mRNAs that are important for development [9,128]. GG provide germ cell identity by controlling translation via the repression of mRNAs that control the fate of somatic cells [37].

## 6. Zebrafish GG

Zebrafish (*Danio rerio*) is one of the most common model organisms with an inherited (preformative) type of PGC determination [18]. The germline cells are determined by maternal factors transmitted to the embryo. In Zebrafish, RNPs containing RNAs for all known germ plasm factors, which are important for PGC determination (e.g., *nanos, dazl*, and *vasa*), localize to the same structure during oogenesis. This is the mitochondrial cloud or the Balbiani body (Bb), a phase-separated structure that is conserved among vertebrate oocytes. It is a large aggregate of organelles found in the oocytes of many species that translocates from a location near the nucleus to the vegetal pole during oogenesis (see also Section 7 and Section 9) [18,43]. The vertebrate animal-vegetal axis is established during oogenesis, whereas the anteroposterior and dorsoventral embryonic axes arise after fertilization. Oocyte polarity is a prerequisite for determining the prospective embryonic axes and germ cell determination in vertebrates with the inherited determination of germ line. Oocyte polarity is initially marked by the asymmetric distribution of organelles, proteins, and mRNAs [19,137,138,139]. Proper regulation of the Bb development is essential to establish the animal-vegetal axis and transfer RNAs and proteins to the vegetal pole.

The Bb is a transient structure assembled in primary oocytes and disassembled thereafter. In Zebrafish oocytes, the Bb assembly in stage Ia (zygotene) primary oocytes and stage Ib (diplotene of meiosis I) requires the protein Bucky ball (Buc) [28,80]. Zebrafish mutant for Buc fails to assemble the Bb or localize mRNAs on the vegetal pole in the oocyte [12]. Additionally, at the animal of wild-type oocytes, a single somatic cell forms the micropyle and channel on the eggshell required for fertilization. The *buc* mutant eggshells have excess micropyles, which leads to polyspermy [80]. A protein Tdrd6a, which contains a multitudor domain, takes part in the Bb aggregation through its interaction with Buc. The inhibition of this regulatory interaction has been linked to significant defects in germ cell development [82]. The Bb disassembly at stage II has been found to be mediated by microtubule actin crosslinking factor 1 (Macf1), a giant multi-domain cytoskeletal linker protein that localizes to the Bb [81].

The Bb is a place of sequestration of germplasm RNAs, such as *vasa, nanos1, and dazl*, along with different RNA-binding proteins, such as Hermes, which is common between both Xenopus and zebrafish Bb [12]. Three pathways that localize RNAs to the Bb are known in vertebrate oocytes: transit through the Bb pathway, utilization of the ‘late vegetal pathway’, and an animal pole transport pathway [14,139].

Besides the Bb, nuage-like structures have been also observed during *Danio* embryogenesis. In fertilized eggs, the nuage structures (1 mkm in diameter) were found near the actin cortex. During the first division, they aggregated into larger complexes that remained in close contact with microtubules and mitochondria. In the blastomeres of the four-cell embryo, patches of nuage were visible in the vicinity of the cleavage furrow [13]. These nuage-like structures are transported to the distal ends of the cleavage furrows by actin microtubules [13], where they participate in germplasm determination. The process results in the determination of four cells with nuage-like structures in the 32-cell stage: blastula [140]. These nuage-like structures are built on homotypic RNP clusters, containing RNAs such as *dazl, dnd, nos-3, rgs14a, and vasa* [83]. The role of *vasa* mRNA in asymmetric segregation is especially important in distinguishing the germline cells from the somatic lineage in cleavage-stage embryos [13]. Segregation of *vasa* mRNA changes from asymmetric to symmetric in the late-stage blastula (spherical). It is linked to RNP dispersal in the cytoplasm of PGCs. This is followed by PGC proliferation and perinuclear localization of Vasa protein [13]. A recently discovered germplasm fragmentation step that precedes the RNP dispersal might contribute to an increase in the pool of germ plasm-carrying cells, presumably PGC [83].

## 7. Xenopus GG

**Figure 3:**
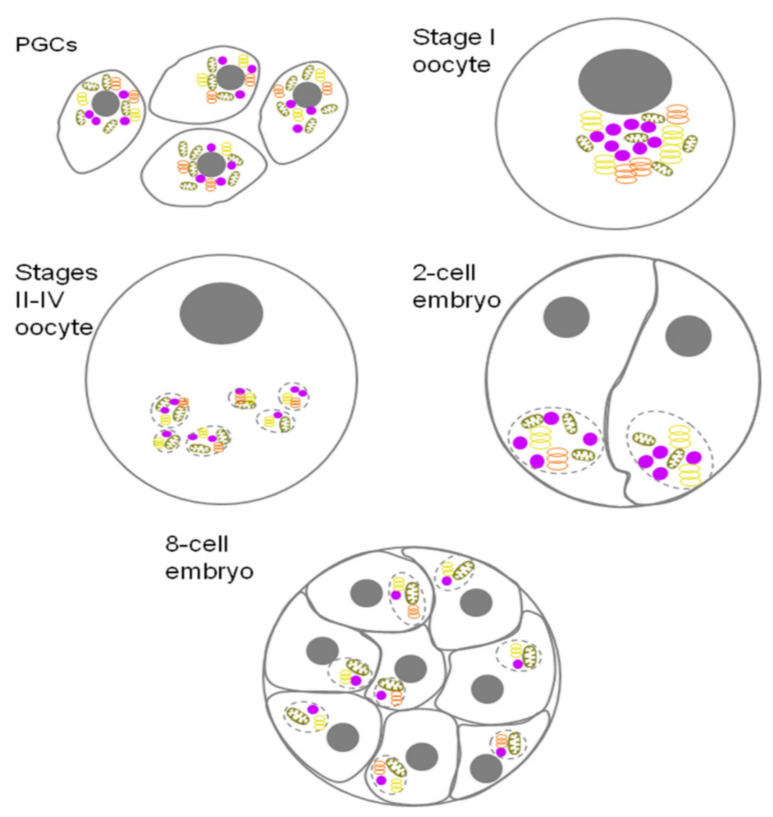
The Balbiani body (Bb) through *Xenopus* oogenesis. In late blastula and gastrula, PGCs are detectable within the embryonic endoderm. The PGCs (PGCs in the image) contain nuage nearby nuclei and mitochondria. During oogenesis, beginning from Stage (I) (shown in the image), a spherical structure that is in contact with the oocyte nucleus and mitochondria is revealed—the Bb [142]. During stage I and the transition into stage (II), the oocyte is arrested in the diplotene of meiosis I. During the subsequent progression through oogenesis, there is a phase of massive growth to accommodate the accumulation of yolk and synthesis of proteins and RNAs, as well as a rearrangement of cytoskeleton. Stages II–IV, shown in the drawing—between stages II and IV of oogenesis, the Bb is divided into ‘islands’ that move towards the vegetal cortex. ‘Islands’ anchored at the top of the vegetative pole of stage VI oocytes are called ‘germplasm islands’. With the onset of embryonic development, at the 2-cell stage, the comonents of the ‘islands’ are divided between the blastomeres of the vegetative pole (‘2-cell embryo’ in the drawing). After fertilization and during early cleavages, ‘germplasm islands’ coalesce into larger aggregates at the vegetal apexes of vegetal blastomeres. (‘8-cell embryo’ in the drawing’). Later, the islands are fragmented again and split between the PGCs. When the PGCs move towards the gonadal bud, the islands substance changes localization to perinuclear, giving rise to nuage. The GG and germ plasm islands are fragmented again and segregate into blastomeres. Germ cells are outlined with grey, nuclei are shown as closed grey circles. Germ granules (closed purple circles). Mitochondria (brown ellipse). Endoplasmic reticulum (ER) (orange rings). Golgi (yellow rings). Germplasm islands are highlighted by a dashed line. Some stages are omitted for better presentation.

*Xenopus* oogenesis has features that distinguish it from other animals. For a long period of time, GGs are incorporated into large, subcellular structures known as the Balbiani body (Bb) (Figure 3). The Bb is a transient structure, as it only exists in the dormant oocytes and disperses once the oocyte is activated (it has also historically been called the yolk nucleus, yolk nucleus complex, nuage body, Dotterkern, and mitochondrial cloud (MC) [25,141].

For many years, the Bb in *Xenopus* has been referred to in most studies as MC [14]. At stage I of the frog oocyte development, the Bb has a spherical structure of about 40 μm in diameter, adjacent to the oocyte nucleus [142]. The Bb is always localized near the prospective vegetal pole of the oocyte, being a marker of polarity. At this stage, the Bb contains approximately 500,000 mitochondria. This observation explains the historical term “mitochondrial cloud”. Mitochondria within the Bb differ in their morphology, enzyme activity and replication properties from mitochondria in other areas of the ooplasm. Thus, the Bb in *Xenopus* contains a germline-specific subset of mitochondria [143]. Other components of Bb are rough ER, numerous Golgi complexes and GG, which are concentrated closest to the pole region. The group headed by Prof S. M. Bilinski termed the region as the messenger transport organizing center, METRO [14]. There are about 700 germinal granules, with diameters ranging from 50 to 2000 nm, within the METRO [15]. During the growth of oocytes, between stages II and VI [142], the Bb is fragmented into many small “islands” that contain all Bb components, including the GG and mitochondria, and moves towards the vegetal pole using an unknown mechanism. ‘Islands’ that are located at the apex of the vegetal pole in the VI stage oocytes are often referred to as ‘germ plasm islands’. By this time, they contain hundreds of small GG (250–500 nm in diameter), numerous mitochondria, Golgi vesicles, and cisternae, as well as ER [15]. With the onset of embryonic development, the components of the islands are divided between the blastomeres of the vegetal pole. The germplasm islands of an eight-cell embryo contain about 80 large (2000 nm in diameter) GG located between mitochondria [12]. Ultimately, the islands are fragmented again and divided between PGCs. When the PGCs move towards the gonadal primordium, the GG substance changes its localization to perinuclear, giving rise to nuage, which was later called mitochondrial cement in *Xenopus* [144]. 

### Xenopus Bb Composition

The Bb is a membraneless condensate of mitochondria, endoplasmic reticulum, Golgi apparatus, proteins, and RNA that break apart when the oocyte begins maturation. The Bb disassembly releases the organelles into the cytoplasm with most of the content appearing at the vegetal pole of a mature egg [145]. It can be isolated manually [146] providing opportunity for biochemical and physical studies. The Bb is spherical in oocytes of many, but not all, species [147]. The spherical shape of other biocondensates (for example, the nucleolus and P-granules) is due to surface tension. Therefore, it seemed logical to assume that the Bb can have the properties of a liquid structure. However, mechanical isolation of the Bb from *Xenopus* oocytes has proven that they behave like solid bodies with a stable structure [97]. Previous electron microscopic studies of human oocytes have also shown the presence of fibrils in the Bb, indicating the ‘solid’ amyloid-like nature of the structure [21]. One of the possible explanations for the spherical shape of the Bb is that this initially liquid formation quickly transforms into ‘solid’ amyloid-like structures. The systematic biochemical analysis of the Bb was carried out for *Xenopus laevis* oocytes [97]. The authors demonstrated that the protein XVelo is the most abundant protein in the *Xenopus* Bb. XVelo is a *Xenopus* homolog of the Buc protein of *Danio*. The gene, coding for this protein, is lost in mouse but has been identified in human and other mammals [148]. Notably, mouse oocytes do not contain conventional Bbs. The structures described previously as Bbs are now considered an unusually shaped Golgi apparatus [149]. XVelo is a protein that contains an IDR and a prion-like domain (PLD) at the N-terminus, as well as a positively charged C-terminus that binds to RNA. When this protein is expressed in vitro, ‘solid’ structures are formed, similar to amyloid fibrils, which in turn are capable of self-organization [97]. These aggregates are stained with Thioflavin T, a dye that marks amyloid-like fibers, as well as the Bb in *Xenopus* oocytes [97]. XVelo-GFP localizes in the Bb after injection into oocytes and fills in the spaces between mitochondria. Apparently, this protein forms a stable matrix in the Bb. This finding, combined with the data about a very high local concentration of XVelo, which exceeds 500 mM, indicates that XVelo acts as a structural adhesive that holds the organelles together in the Bb [97]. This is consistent with the modern concept that GG and Bb are biomolecular condensates formed via the phase transition mechanisms [1,2]. The idea of an amyloid-like basis for the Bb also correlates well with the possible function of this structure, which is to maintain a low activity of mitochondria and maternal RNAs, so that oocytes could overcome the long waiting time of the resting stage. The assembly mechanism of ‘solid’ Bbs, in comparison with liquid drops of GG, is based on the exclusion of a large amount of water from the structure. The amyloid state is achieved by a combination of many β-sheets of proteins through the formation of hydrogen bonds between them [150]. In addition to self-aggregation, XVelo interacts with proteins Tdrd6 and Rbpms2 [12,82]. Tdrd6 is a protein containing the Tudor domain and is a component of the Piwi pathway. However, Tdrd6 is not required for the piRNA pathway, but instead, is important for PGC formation and the structural integrity of the Bb.

In *Xenopus*, the Bb is also needed to establish the animal-vegetal axis during oogenesis by the asymmetric distribution of organelles, proteins and mRNAs within oocytes [138,139]. This fact distinguishes the functions of Xenopus’ and Danio’s Bbs from Bbs of mouse and human. In the latter species, asymmetry is not required at all stages of oogenesis, and mouse and human Bbs are involved rather in protein and mRNA sequestration and retroposon inactivation than in establishing asymmetry [113,138,151]. The Bb is considered now as a site of early pathway RNA localization during stage I of *Xenopus* oogenesis [152]. The distinct stages of *Xenopus* oogenesis are marked by the developmentally staged localization of maternal RNAs. There are two main ways of ensuring RNA localization at the vegetal pole in *Xenopus*. (1) METRO or early RNA localization mechanism. Due to this mechanism, the RNAs of *Xcat2, Xdazl, Xpat, Xlsirts, Xwnt11, DEADSouth, Fingers, XFACS, and Xtox1* move to the vegetal pole [14,15,85,86,87,88,92,93,94,152]. (2) Late or Vg1 pathway for microtubules-mediated RNA movement to the vegetal pole. This pathway starts after all the mRNAs destined to the Bb enter it. RNA transported by the Vg1 pathway along the microtubules is not localized in the Bb [91]. The Vgl pathway provides a transport mechanism to the vegetative pole for many specific transcripts, including VegT, an induction factor of the mesoderm of the embryo. There is also a mixed pathway for moving specific transcripts, such as Hermes RNAs, which play an important role in the development [92].

Among the RNAs that enter the Bb via the METRO mechanism, *Xcat2* was identified, which encodes a protein containing a Nanos-like zinc finger motif [91]. Nanos is one of the classical determinants of germ line cells. RNA *Xdazl* was also identified, which is also involved in the formation of the germ line cells [84] and is present in nuage up to stage I of oocyte development. Xdazl is localized in the cytoplasm between GG in more mature oocytes and in embryos [15]. Among other RNAs, transportation along the METRO cascade is also characteristic of *Xpat* and *Xlsirts* RNAs, the latter RNA is noncoding and belongs to the family of highly repetitive tandemly organized RNAs [85,86]. These RNAs are localized in the center of the nuage in oogonia and in the cytoplasm between GG in embryos [15]. *Xenopus* Bbs also contain a homologue of the Vasa protein (another germ line cells marker)—XVLG1 [95]. In addition, *DEAD South* mRNA was identified, which encodes Vasa-linked RNA helicase, closely associated with EIF4A [87], and Fingers, which encodes a protein homologous to the transcriptional repressor—protein Kox1 and contains a Kruppel-like zinc finger motif [15].

The obtained data show that proapoptotic proteins Bax and p53 are sequestered in the oocyte I–early II stage nucleus (that means the exclusion of the proteins from the cytoplasm) and antiapoptotic protein Bcl-xL is sequestered in the cytoplasm and highly enriched in the METRO where it is colocalized within GGs [96] 

*Xenopus* oogonia and I and early II stage oocytes have no indication of apoptosis even during apoptosis-inducing stress [96,144]. Meanwhile, fully grown Stage VI *Xenopus* oocytes seem to be able to undergo apoptosis: the expression of exogenous Bcl-xs induces oocyte death by a caspase-dependent mechanism and is inhibited by coexpression of exogenous antiapoptotic Bcl-xL [153]. The absence of apoptosis in early oogenesis in *Xenopus* is due to the differential sequestration of antiapoptotic and proapoptotic factors because the progress and the outcome of the apoptotic pathway depend on the relative concentration and interaction (which occurs in the cytoplasm) between antiapoptotic and proapoptotic proteins [96]. The biological sense of this in agreement with the theory of Krakauer and Mira [154] based on the evolution way of *Xenopus*. Compared with mammals, *Xenopus* produces a large number of oocytes with a large number of mitochondria. The massive death (atresia) of the germ cells in mammal ovaries is usually interpreted as a developmental solution to the accumulation of detrimental mutations in female germ cell mitochondria. In contrast, species that produce large litters can procreate inferior (containing defective mitochondria) progeny, because the high number of offspring ensures that some high-quality survivors live to reproduce. Therefore, it may be evolutionarily advantageous to shift the timing of elimination of defective mitochondria from the early oogenesis stage to the late oogenesis or embryonic stage [96].

Thus, in *Xenopus*, the Bb is a membraneless ‘solid’ biocondensate of amyloid nature. Its main functions seem to be the identification of the PGC lineage and the spatial sequestration of some mRNA organelles until the oocyte maturation begins.

## 8. Birds

Recent data suggest that a subset of Reptilia (including avian species according to the current classification), specify germ cells via an inherited mechanism (preformation), whereas at least some non-avian reptiles use the inductive mechanism [16,17,18]. In 2000, Tsunekawa et al., described chicken Vasa homolog (CVH) [16]. The authors provided evidence that CVH protein was colocalized with spectrin and mitochondrial clouds in growing oocytes CVH in a characteristic globular shape structure in oocytes. The exogenous expression of *Cvh* gene, combined with appropriate culture conditions, induces cESC reprogramming towards a germ cell fate [155]. The finding confirmed the presence of germ plasm with maternally inherited factors in chicken oocytes. The maternally inherited factors are distributed asymmetrically between blastomeres. However, it is not clear yet whether the asymmetry is involved in the asymmetry of the first two cleavages in chicken embryo [156]. It was demonstrated recently that the chicken homolog of deleted in azoospermia-like (cDAZL) is involved in germ line specification and its localization in the oocyte’s central part leads PGCs to be formed in the center of an embryo [17]. The proteins of this family are crucial for PGC formation both in organisms with inherited and inductive mechanisms of PGC determination [157].

## 9. GG in Mammalian Oocytes

**Figure 4:**
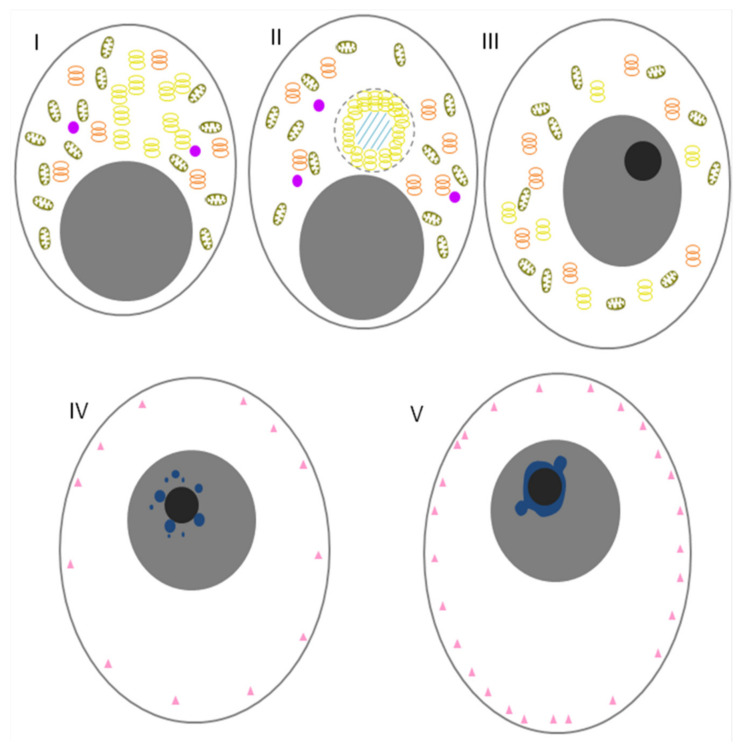
Schematic diagram of the Golgi ring of mice. (**I**) An oogonium within a germline cyst. (**II**) An oocyte within a primordial follicle shows a well-defined Golgi ring surrounding the mitochondrial exclusion zone (MEZ). (**III**) An oocyte within a primary follicle with mitochondria and ER, evenly distributed throughout the cytoplasm. (**IV**) NSN GV oocyte. (**V**) SN GV oocyte. Germ cells are outlined with grey, and nuclei are shown as closed grey circles. Germ granules/nuage (closed purple circles). Mitochondria (brown ellipse). Golgi (yellow rings). Endoplasmic reticulum (ER) (orange rings). Subcortical aggregates (pink triangle). Condensed chromatin (Blue). In II, the Golgi ring is marked with a dashed line. MEZ is marked with hatching.

It was believed for a long time that mammalian oocytes were nonpolar cells that did not contain any structures corresponding to GG, and that the PGC lineage was formed as a result of induction by the BMP and WNT signalling pathways [158]. However, it turned out that the oocytes of most marsupial mammals, rats, hamsters, guinea pigs, rabbits, goats, and primates, including humans, were polarized to some degree and contained Bbs, a component of which was nuage. The detected structures were similar in their ultrastructural and dynamic characteristics to the Bb [137]. Until the last decade, mice stood out among all mammals; they were thought to be an exception and their oocytes did not contain any Bb-like structure [137,138]. Pepling and colleagues were the first to describe the presence of Bbs in neonatal mouse oogonia and oocytes of primary follicles. The authors showed that the Bb was a temporary structure that was disassembled during late oogenesis. Three-dimensional reconstruction of the Bb structure showed that the oocytes of newborn mice were not only asymmetric but also polar, like early *Xenopus* oocytes [159]. Despite this similarity, the overall structure of the Bb in a mouse differs from that of *Xenopus*: in mouse, the Bb does not contain mitochondria but is surrounded by a Golgi complex while in Xenopus, the Bb play role in forming a germline-specific subset of mitochondria [19,143]. Therefore, the structure was sometimes referred to as a Golgi ring (Figure 4). However, in a study that has been carried out recently on live oocytes by Dr. Elvan Böke’s team, the mouse Bb-like structure has been proven to be different from human and frog Bbs. Moreover, the authors suggested the structure could not be classified as a Bb [149]. The main difference was the absence of the matrix of IDR proteins (like XVelo in *Xenopus*) that holds together the Bb components. Another important finding arguing against the Golgi ring as the Bb is the fact that the Golgi ring formation is reversible, and not linked to the dormancy status of the oocyte [149]. It is not known whether the Golgi complex is involved in the polarization of any molecule or organelle at the mouse oocyte’s vegetal pole. It is possible that the vesicles of the Golgi complex in the Bb contain certain components of the extracellular matrix of zona pellucida [160,161]. If this is the case, then it is possible that the Bb forms the temporal polarity of the mouse oocyte, which is transformed into the polarity of the zona pellucida due to the secretion of the zona pellucida components from the Golgi complex [159]. Another assumption about the Bb function is based on a possible relationship between the cisterns of the Golgi complex at the vegetal pole and asymmetric meiotic division. It has been shown that GM130, a resident protein of the Golgi complex, binds to the meiotic spindle and plays a key role (possibly due to its interaction with proteins of the MAPK cascade) in the organization and polar migration of the spindle apparatus during isolation of the first polar body in a maturing mouse oocyte [162]. It has also been suggested that the aggregation of mitochondria near the Golgi apparatus facilitates their perception of stress signals, which ultimately leads to the elimination of damaged mitochondria and prevents their inheritance by the offspring [163]. Possibly, the polarity of mouse oogonia is associated with the organization of the Bb around the classical centriolar centrosome, which is present at this stage. During the development of oocytes, centriolar centrosomes become acentriolar ones, which can cause a loss of polarity.

**Figure 5:**
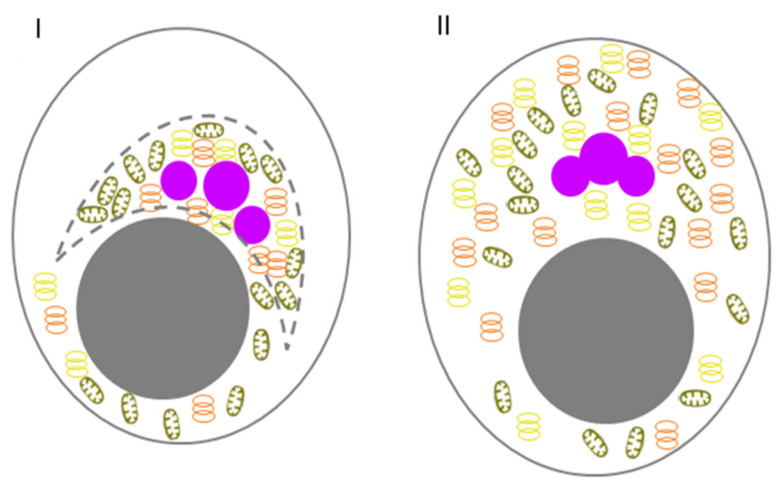
Schematic diagram of a Balbiani body in human. (**I**) An oocyte within a primordial follicle showing a Balbiani body. (**II**) An oocyte within a primary follicle. Germ cells are outlined with grey, and nuclei are shown as closed grey circles. Germ granules (closed purple circles). Mitochondria (brown ellipse). Golgi (yellow rings). Endoplasmic reticulum (ER) (orange rings). In (**I**), the Balbiani body is marked with a dashed line.

In contrast, Bbs in human oogonia (Figure 5) are highly enriched in mitochondria [21]. Both in live and fixed oocytes, the Bd is crescent-shaped and attached to the nucleus [149,164]. Its structure has more similarity to the structure of the *Xenopus* Bb than the mouse Golgi ring.

The molecular composition of the mammalian Bb is being actively studied now, mostly in mice (though the structure is not fully identical to a classical Bb) and humans. MVH, a homologue of Vasa in mice, was found in oocytes, being evenly distributed in the cytoplasm, and it was specifically localized in the perinuclear chromatoid body in spermatocytes [100]. In humans, a homologue of MVH -a protein DDX4 is associated with the Bb in primordial and primary follicles [106]. In mice, MVH is associated with many proteins, including PADI6 and NLRP5, elements of the oocyte subcortical maternal complex (SCMC), and the NLRP5 and FILIA proteins [101]. All of these proteins are proteins of the cytoplasmic lattices—a keratin-containing fibrillar matrix found in the early stages of oocyte growth, which persists until the blastocyst stage [102] in primordial follicles, MVH and cytoplasmic lattice proteins, together with piRNA form RNP complexes, which perform two functions: they play a role in the regulation of retrotransposons and also participate in the sequestration of vital maternal transcripts (for example, MTA/Dnajc11 or MTA/Spin1) [99]. The late translation begins when the expression of nuage proteins stops, and the amount of MVH decreases, RNP complex dissociates, and proteins of the cytoplasmic lattices move to the perinuclear region. Associated RNAs are released and can be translated [99]. Though the Bb formation in mice is under question now, nuage existence is not doubted. Both in human and mouse, the MVH protein and its human homologue—DEAD-box RNA helicase DDX4 are considered as marker proteins for nuage, which are liquid biocondensates, unlike the Bb [42]. Nuage and the Bb are dissociated early in oogenesis. Nevertheless, we revealed RNP granules accumulated in the ooplasm of fixed oocytes during GV-MI that contained an IDR-containing helicase DDX4 [165]. DDX4 has extended N- and C-terminus, which are supposed to contain IDR [166]. The N-terminus of DDX4 spontaneously self-associates both in cells and in vitro. Condensed protein droplets have a liquid interior. Two highly conserved regions in the Ddx4 sequence were identified that allow the formation of liquid droplets: repeating blocks of 8–10 amino-acid residues, with alternating opposite charges and a large number of phenylalanine—glycine FGG and arginine; glycine RGG repeats within positively charged blocks. These regions are also found in other IDR proteins. The processes of condensate regulation are similar. Nuage phase separation is hindered by post-translational modifications, for example, methylation of arginine domains in DDX4 [167]. In some works of recent years, a fundamentally new mechanism of condensate regulation has been proposed. The reentrant phase transition theory is based on the idea that low RNA concentrations promote the formation of condensate droplets, while higher RNA concentrations lead to their dissolution through a charge inversion mechanism [168]. It was experimentally shown that DDX4 drops differentially solubilize nucleic acids. Single-stranded DNA is concentrated inside the nuage, while double-stranded DNA is largely excluded from the droplets [167]. This is consistent with RNA processing functions that are inherent in many membraneless organelles.

Unlike *Xenopus*, in human oogenesis, the proapoptotic protein BAX has a constant localization throughout the different stages of folliculogenesis, from dormant primordial follicle reserve to antral follicles entering cyclical follicular growth. The constant expression of *BAX* has also been reported to occur throughout fetal life, from the early proliferative stage of primordial germ cell and oogonia, during the entrance to prophase I at mid-gestation, and from primordial follicular assembly to the end of gestation [106,107,169]. On the other hand, antiapoptotic BCL2 protein was not detectable in dormant primordial follicles and primary follicles leaving the resting reserve. It became detectable in the somatic stratum from secondary follicles, as well as preantral and antral growing follicles, co-existing with BAX protein. In fetal life, BCL2 also shows a time-restricted pattern of expression occurring in oogonia at the proliferative stage from gestation week 12 to 18, before primordial follicle assembly [106,107]. BCL2 and BAX proteins in a normal human infant and pubertal ovary behave as in fetal life, as far as the oocyte remains in the primordial resting reserve, whereas in follicles entering the growing pool gene expression moves from the germ cell to granulosa cells, with an expression pattern comparable to the adult ovary [170]. Unexpectedly, in adult ovaries, BCL2 protein was detectable throughout folliculogenesis, including primordial and primary follicles of the germinal reserve. The expression of BCL2 in the primordial reserve, both in patients that had or had not received chemotherapy, rules out the possibility that BCL2 expression may be linked to a chemo-treatment response [171].

Knudson et al. [172] demonstrated that, in mice, BAX deletion resulted in the appearance of unusual atretic follicles with their granulosa cells seemed unable to activatee apoptosis. Evidence has been recently obrained that death mechanisms such as caspase-independent cell death and autophagy may be acting in the mammalian ovary [173]. BCL2 plays essential roles in the crosstalk between autophagy and apoptosis [174]. Even if BCL2 suppresses autophagy by binding to Beclin I protein, crosstalk between both mechanisms is not so simple because autophagy can help cell survival by suppressing apoptosis or lead the cell to death in collaboration with apoptosis [174].

BAX and BCL2 proteins move from the germ cell to the somatic stratum when primordial follicles leave the resting reserve to enter the growing follicular pool. The same pattern was also found in adult ovaries [170]. According to the available data, the main functions of nuage in mammalian oogonia are suppression of retrotransposon transcription, inactivation of mRNA, and interaction with proteins of the cytoplasmic lattice [99]. However, unlike male gametogenesis, the inactivation of the protein components of oogonia nuage does not lead to infertility [175]. Thus, it is most likely that, in mammals, GG are not so much involved in the process of PGC determination, as they are involved in the inactivation of retroposons and associated processes of chromatin remodelling.

## 10. Conclusions

Research in recent years has expanded our knowledge of the molecular mechanisms underlying the assembly and functioning of biomolecular condensates. Many of these structures, including GG, are formed as a result of a ‘liquid-liquid’ phase transition caused by interactions of multivalent molecules. Some condensates, such as the Bb, are capable of forming denser structures by the mechanism of ‘liquid-solid’ phase transition. The composition of GG may vary, but there is a set of markers for them. It includes both proteins (families Vasa, Tudor, Nanos, Dicer, and Argonaute) and RNA (mRNA, piRNA, and mi/siRNA), which allows for the differentiation and classification of various structures. The main functions of GG in most animals are the formation of a pool of germline cells and spatial sequestration of maternal RNA and proteins. In addition, GG are involved in the control of mRNA translation and expression of individual genes, including the inactivation of retroposons. These functions can be established by GG, due to their liquid droplet characteristics, which allows molecules to concentrate in condensates, thus maintaining continuous exchange with the environment, which is not hindered by a membrane barrier. However, the properties of biocondensates also determine their functional limitations. Small molecules, such as ions, are difficult to keep inside the condensate. In addition, the absence of a membrane makes it difficult to maintain a stable pH in them. Therefore, the two ways of cell organization—membrane organelles or biomolecular condensates—complement each other and, together, provide maximum opportunities for organizing cellular contents.

Despite our progress in understanding the role of biocondensates in animal oogenesis, the details of biocondensates assembly and functions are not clear yet. Further studies will help to increase our understanding of germ line cells determination, differentiation, and maturation, thus providing us with new tools for infertility therapy.

## Figures and Tables

**Table 1:** Diversity of GG in different animal species.

Species	Type of Cells	Stage of Development	Historical Name of the Structure	Modern Name of the Structure	Reference
*Caenorhabditis elegans*	Cells of germ line at all stages of development	Four larval stages and an adult	P-granules	P-granules/nuage	[6,7]
*Drosophila melanogaster*	In oocytes and nurse cells	All stages of oogenesis	Sponge bodies	GG	[8]
	In nurse cells	All stages of oogenesis	Nuage	Nuage	[9,10]
	In oocytes and embryos	Late stages of oogenesis and early embryogenesis	Polar granules	GG	[9,10]
*Danio rerio*	Primary oocytes	From stage Ia (zygotene) to stage Ib (diplotene) of oocytes development	Nuage/intermitochondrial cement	Bb	[11,12]
	Embryos	From zygote to 32-cell embryo	GG as part of germ plasm islands	Nuage-like structures	[12,13]
*Xenopus laevis*	PGC and early oocytes	From PGC to I stage of oocytes development	Mitochondrial cloud and subsequent stages of its development	Nuage	[14]
	Oocytes	From I to Vl stage of oocytes development	GG as part of Bb	GG	[14,15]
	Late oocytes and early embryos	From Vl stage of oocytes development to 8 cell embryo	Small GG as part of germ plasm «islands»	GG	[14,15]
	Embryos	From eight cell embryo to PGC	GG as part of germ plasm ‘islands’	GG	[15]
*Gallus gallus*	Oocytes	Oocytes	Mitochondrial cloud	CVH-positive structures	[16,17,18]
	Early embryos	From embryo stage I to stage X	Mitochondrial cloud	CVH-positive structures	[16]
*Mus musculus*	Oogonia and oocytes of primary follicles	Early stages of oogenesis	Nuage	Nuage	[19]
	Spermatocytes	Spermatogenesis	chromatoid body	Nuage/chromatoid body	[20]
*Homo sapiens*	Oogonia	Early stages of oogenesis	Nuage	Nuage	[21]
	Spermatidsspermatocytes	Spermatogenesis	Chromatoid body	Nuage	[22,23,24]

**Table 2:** Composition of GG in different species.

Species	Modern Name of the Structure	Marker RNA	Marker Protein
*C. elegans*	Nuage/P-granules	mRNA *Nos-2* [35,48] 26G siRNA-3[49] piRNA (21U) [50] 22G-siRNA [51]	ZNFX-1 and WAGO-4 [52] GLH-1, 2, 3, 4 [53] RDE-12 [54] PGL-1, PGL-3 [53,55] LAF-1, VBH-1, CAR-1, CSR-1, ALG-3, ALG-4, and HRDE-1 [56] WAGO-1 [57] MEG-1, MEG-2, MEG-3, and MEG-4 [58] Nanos [35] PUM 2 [33] CGH-1 [59]IFE-1(EIF4E) [60] DCP-2 [61] DCR-1, DRH-3, EGO-1, CSR-1, and PRG-1 [51] PGL-3, MEX-5 И PAR-1 [62]
*Drosophila melanogaster*	GG/sponge bodies	mRNA *Bicoid* [63]	Exuperantia И Me31B [8,63,64] Bruno (in nurse cells), Orb (in oocytes) [8,64,65,66] EIF4e [64] Cup [67] Dcp1 and Dcp2 [68] Nanos [37] PUM 2 [33] α-spectrin [69]
	GG/polar granules		Vasa [70] Oskar [71] Dcp1 И Me31B [64,72] Aubergine [73] Tudor [74] EIF4A [64] PIWI, DICER-1 И dFMRP (fragile X mental retardation protein) [41] α-spectrin [69]
	Nuage	Small interference RNA (siRNAs) [75]	Vasa [70] Ago3 [76] Aubergine [73] Maelstrom, Spindle-E [77] Krimp, Tejas, and PAPI [75,78,79] α-spectrin [69]
*Danio rerio*	Bb	*Vasa, Nanos1, Dazl* [12]	Buc [28,80] Vasa [13] Hermes [12] Macf1 [81] Tudor (Tdrd6) [82]
	Nuage-like structures	*Dazl, Dnd, Nos-3, Rgs14a, Vasa* [83] *Brul* [43]	Nanos 1 [34]
*Xenopus laevis*	Nuage/mitochondrial cloud	*Xdazl* [84] *Xpat* [85] *Xlsirts* [86] *DEADSouth* [87] *Fingers* И *XFACS* [15] *Syntabulin* [88]	XALK4 [89] EF-1 [90] Tudor (Tdrd6) [12] Nanos, Pumilio [37]
	GG	Xcat2, Vg1 [91] Xdazl, Xpat, Xlsirts [15] Hermes, Nanos1, Vasa and Dazl [92] Xwnt11, VegT [14] Xotx1 [93] mRNA glutamate receptor interacting protein 2a (Grip2a) [94]	XVLG1 [95] EIF4A [87] Kox1 [15] Bcl-xL [96] XALK4 [89] XVelo [97] Rbpms2 [82] Tudor (Tdrd6) [12] Macf1 [81] Nanos, Pumilio [37]
	Small GG as part of germ plasm islands	Xcat2, Vg1 [91] Xdazl, Xpat, Xlsirts [15] mRNA Glutamate receptor interacting protein 2a (Grip2a) [94] RNA Syntabulin [98]	XVelo [97] Tudor (Tdrd6) [12] Nanos, Pumilio [37]
*Gallus gallus*	CVH-positive structures		CVH (chicken Vasa homolog)[16]
*Mus musculus*	Nuage	mRNA *Dnajc11, Spin1* [99], piwiRNA [99]	MVH [100] PADI6, NLRP5 И FILIA [101,102] DDX6, CPEB, YBX2 (MSY2), (Exon junction complex, EJC), DCP1A [103] TDRD1 [104] Nanos1, Pumilio2, Gemin3 [36] Maelstrom [105]
*Homo sapiens*	Nuage		DDX4 [42] BAX (oogony and oocytes) [106] BCL2 (only oogonia) [106] Mcl-1 [107] Nanos, Pumilio И Gemin3 [36]

## Data Availability

The study reports no data.
